# Searching for putative avian malaria vectors in a Seasonally Dry Tropical Forest in Brazil

**DOI:** 10.1186/s13071-016-1865-y

**Published:** 2016-11-16

**Authors:** Francisco C. Ferreira, Raquel A. Rodrigues, Yukita Sato, Magno A. Z. Borges, Érika M. Braga

**Affiliations:** 1 Departamento de Parasitologia, Instituto de Ciências Biológicas, Universidade Federal de Minas Gerais, Av. Antônio Carlos 6627, Belo Horizonte, MG 31270-901 Brazil; 2 Laboratory of Biomedical Science, Department of Veterinary Medicine, Nihon University, Kameino 1866, Fujisawa, Kanagawa 252-0880 Japan; 3 Laboratório de Controle Biológico, Centro de Ciências Biológicas e da Saúde, Universidade Estadual de Montes Claros, Campus Universitário Professor Darcy Ribeiro, Montes Claros, MG 39401-089 Brazil

**Keywords:** *Plasmodium*, *Haemoproteus*, Haemosporidia, Vectors, Culicidae, *Mansonia*, Ecological succession, Habitat modification

## Abstract

**Background:**

Haemosporidian parasites of the genera *Plasmodium* and *Haemoproteus* can have detrimental effects on individual birds and populations. Despite recent investigations into the distribution and richness of these parasites and their vertebrate hosts, little is known about their dipteran vectors. The Neotropics has the highest diversity of mosquitoes in the world, but few studies have tried to identify vectors in this area, hampering the understanding of the ecology of avian malaria in the highly diverse Neotropical environments.

**Methods:**

Shannon traps and active collection were used to capture 27,110 mosquitoes in a Seasonally Dry Tropical Forest in southeastern Brazil, a highly endangered ecosystem.

**Results:**

We screened 17,619 mosquito abdomens from 12 different species and several unidentified specimens of *Culex*, grouped into 1,913 pools, for the presence of haemosporidians. Two pools (out of 459) of the mosquito *Mansonia titillans* and one pool (out of 29) of *Mansonia pseudotitillans* were positive for *Plasmodium* parasites, with the detection of a new parasite lineage in the former species. Detected *Plasmodium* lineages were distributed in three different clades within the phylogenetic tree revealing that *Mansonia* mosquitoes are potential vectors of genetically distant parasites. Two pools of *Culex* spp. (out of 43) were positive for *Plasmodium gallinaceum* and closely related lineages. We found a higher abundance of these putative vectors in pasture areas, but they were also distributed in areas at intermediate and late successional stages. One pool of the mosquito *Psorophora discrucians* (out of 173) was positive for *Haemoproteus*.

**Conclusions:**

The occurrence of different *Plasmodium* lineages in *Mansonia* mosquitoes indicates that this genus encompasses potential vectors of avian malaria parasites in Brazil, even though we did not find positive thoraces among the samples tested. Additional evidence is required to assign the role of *Mansonia* mosquitoes in avian malaria transmission and further studies will add information about evolutionary and ecological aspects of avian haemosporidia and untangle the diversity of their vectors in Brazil.

## Background

Avian haemosporidians are vector-transmitted parasites that can negatively impact natural bird populations by reducing host fitness [1] or by acting as primary or secondary etiological agents in mortality episodes [2]. Two widespread genera of these avian haemosporidians are *Plasmodium* spp. transmitted by mosquitoes (Culicidae) and determined as avian malaria; and *Haemoproteus* spp. transmitted by biting midges (Ceratopogonidae) or louse flies of the family Hippoboscidae [3].

Transmission occurs globally, and host-parasite compatibility can determine the heterogeneous distribution of these parasites [4]. Vector-parasite compatibility [5] and mosquito feeding patterns [6] also play a role in parasite distribution. Nevertheless, haemosporidian vectors have received less attention than their vertebrate hosts, and this is especially true for tropical latitudes. The diversity of avian haemosporidian vectors is higher than previously thought [7], but despite having the highest diversity of mosquitoes globally [8], the Neotropical Region is still understudied [9].

Several studies have revealed a heterogeneous distribution of avian haemosporidians across Brazilian biomes such as the Atlantic [10] and Amazonian Rainforests [11, 12], the Brazilian Savannah [10, 13, 14], and Seasonally Dry Tropical Forests [10]. However, haemosporidian vectors have not been described in these areas, and their importance in determining parasite distributions has, therefore, not been assessed. In this study, we surveyed mosquito populations in Seasonally Dry Tropical Forest (SDTF) fragments at different successional stages aiming to identify putative avian malaria vectors in Brazil.

## Methods

### Study site

This study was conducted at Mata Seca State Park (MSSP), a conservation site encompassing an area of 15,466 ha, located in the River São Francisco valley in southeastern Brazil (14°48′36″–14°56′59″S and 43°55′12″–44°04′12″W). The regional climate is classified as tropical with dry summers (“*As*” category in Köppen’s classification [15]), with an average temperature of 23.4 °C. The dry season starts in May with an average rainfall of 6 mm and the four following months typically receive less than 10 mm of rain. The rainy season starts in October and peaks between December and January, with monthly precipitation around 200 mm [15]. The area has a history of extensive cattle grazing, and approximately 1,525 ha of the MSSP consist of abandoned pasture fields at different successional stages, with the remaining areas considered as mosaics of secondary and primary patches of SDTF. These forests are dominated by deciduous trees that lose up to 95% of leaf area during the dry season [16].

**Fig. 1:**
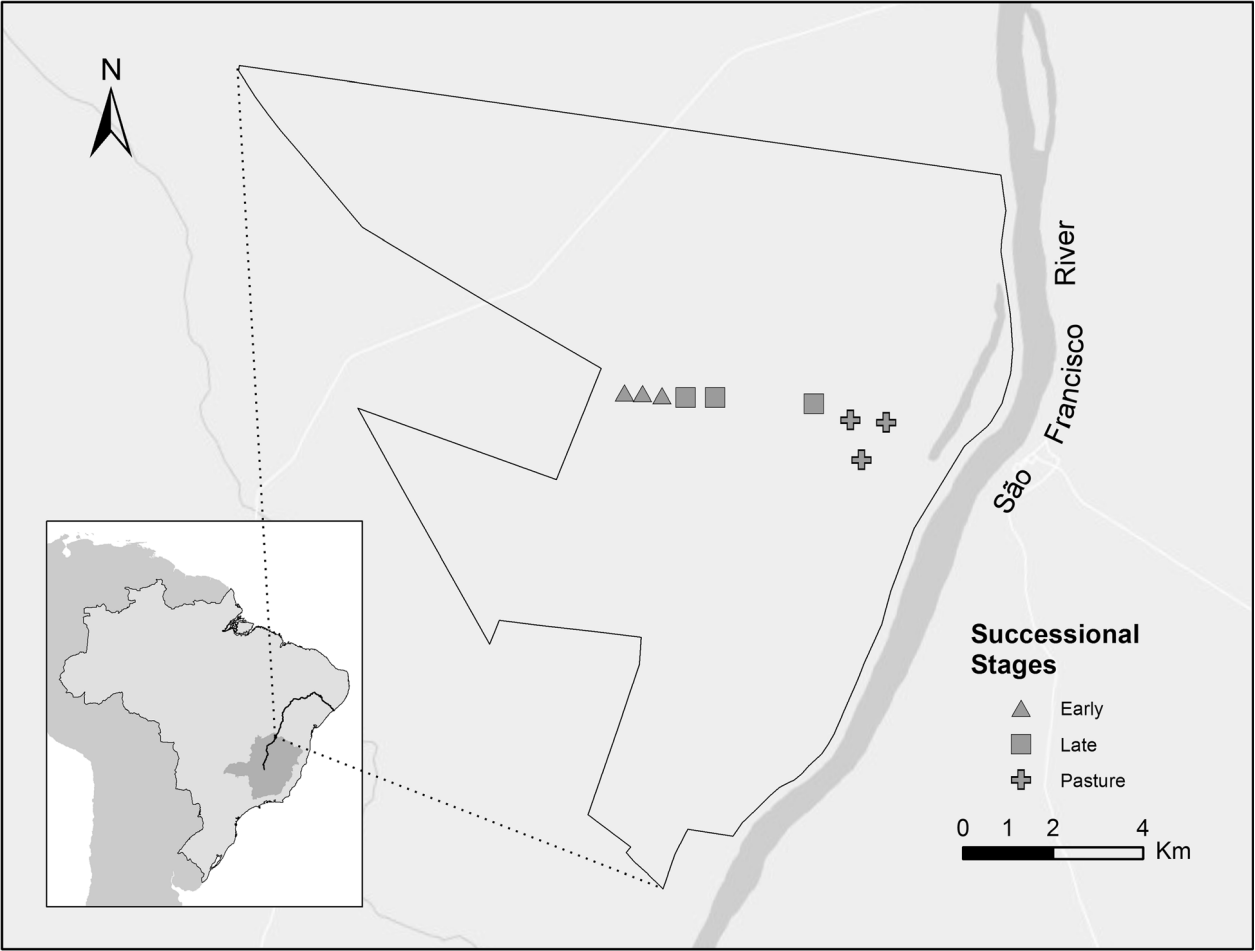
Map of Mata Seca State Park in Minas Gerais, Brazil, showing the sampling areas

We captured mosquitoes in areas representing three different successional stages inside the park (Fig. 1). The area defined here as “pasture” was used for extensive cattle grazing and was abandoned in 2008, five years before the beginning of the present study. This vegetation consisted of exotic grass species, herbs and shrubs, and sparse trees. We defined as “early stage”, a pasture field used for cattle grazing for over 20 years that was abandoned in the year 2000, 13 years before the study. This successional stage was dominated by a single stratum of young trees. Finally, the “late stage” had two tree strata, with tall deciduous trees forming a closed canopy at 18–20 m from ground level. The lower strata consisted of a sparse understory with a low density of young trees and lianas. This area has no record of human intervention for over 50 years. Many temporary lakes are scattered throughout the park, and there is a permanent lake situated at 2 km from the pasture areas.

Sampling was carried out at the end of the rainy season of 2013 (April), at the middle and at the end of the dry season of 2013 (June and September, respectively) and at the peak of the rainy season of 2014 (January). For data comparison, we established three plots within each successional stage, with the same sampling effort for all plots and periods. Distances between plots within the same stage varied from 0.5 to 1.5 km.

### Mosquito collection and processing

Mosquitoes were collected using Shannon traps and by active collection. LED lamps were set up inside the Shannon traps, and all attracted insects were captured using automatic aspirators (made by adapting a fan from CDC-like traps to a PVC tube 15 cm in diameter, sealed by a fine mesh at the bottom) during two hours starting at dusk. These samplings were conducted at the same place and period as bird trapping, so we used automatic aspirators to collect mosquitoes that were attracted by researchers while setting up mist-nets and while taking blood samples from trapped birds. These methods rely on the active capture of mosquitoes by researchers, hence it is biased towards mosquito species that are attracted to humans and that are more active during the day and early night hours. Collected mosquitoes were kept at 4–8 °C at the field site for a maximum of five days and transferred afterwards to a -20 °C freezer. Mosquitoes were identified using reference keys [17, 18] and separated by species, day, method and place of collection. Mosquito genera were abbreviated according to Reinert [19]. Voucher specimens were deposited in the laboratory where mosquitoes were identified (Laboratório de Controle Biológico, Universidade Estadual de Montes Claros, Brazil). Samples were stored in absolute ethanol in 1.5 ml tubes at -20 °C until processing. Legs, wings, and the anterior part of the head of unfed females were removed using microscissors. Subsequently, the thorax was separated from the abdomen for all mosquitoes and each segment was kept in pools of up to 15 individuals (average of nine). Engorged females were kept in individual tubes, without being dissected. The collection of mosquitoes complied with local regulations (Sistema de Autorização e Informação em Biodiversidade - SISBIO license 29899).

### DNA extraction

Genomic DNA from abdomen and thorax pools was extracted using the QIAamp® DNA Mini Kit (QIAGEN, Hilden, Germany) following the manufacturer’s instructions with slight modifications. Samples were disrupted in 80 μl of 1× PBS using sterile pestles with a batery-operated grinder. After that, 100 μl of ATL buffer and 20 μl of proteinase K were added and the samples were incubated at 56 °C for a minimum of 1 h. Thereafter, 200 μl of AL buffer were added and samples incubated at 70 °C for 10 min. To minimize the amount of chitin, the samples were centrifuged at 20,000× *g* for 5 min and the clean supernatant transferred to a new tube. From this step, the manufacturer’s protocol was followed. Genomic DNA from engorged females was extracted using the REDExtract-N-Amp™ Tissue PCR kit (Sigma-Aldrich, St. Louis, USA) following the manufacturer’s instructions.

### Haemosporidian analysis

Abdomen pools were screened for the presence of haemosporidians, and DNA was we extracted and tested from thorax pools corresponding to the positive samples. Engorged females were also tested for haemosporidians. We used a nested PCR that detects *Plasmodium* and *Haemoproteus* in the same reaction and amplifies a segment of their cytochrome *b* gene (hereafter “cyt *b*”). Primers in the first reaction, HaemNFI (5′-AGA CAT GAA ATA TTA TGG ITA AG-3′) and HaemNR3 (5′-GAA ATA AGA TAA GAA ATA CCA TTC-3′) [20], were combined with 1 μl of genomic DNA and the master mix (described below). A 1-μl aliquot of this PCR product was used as a template for the second reaction with the primers HaemF (5′-CTT ATG GTG TCG A-T ATA TGC ATG-3′) and HaemR2 (5′-CGC TTA TCT GGA GAT TGT AAT GGT T-3′) [21]. Both reactions contained 1× buffer, 4 mM of MgCl_2_, 0.3 mM of each dNTP, 1 unit of Taq (TAKARA Ex Taq® DNA Polymerase, Shiga, Japan), 0.4 mM of each primer, and nuclease-free water in 25 μl reaction volumes. DNA extracted from a *Plasmodium*-infected *Culex pipiens pallens* mosquito collected in Japan, and nuclease-free water were used as positive and negative controls, respectively. Cycle conditions followed Hellgren et al. [20], and PCR products were visualized on 1.5% agarose gels stained with ethidium bromide. Positive samples were sequenced bi-directionally with dye-terminator fluorescent labeling through automated sequencing (ABI Prism 3100, Applied Biosystems). The sequences were edited using MEGA 6.0 [22] and compared to the data available in public databases such as GenBank and MalAvi [23]. We considered sequences as different cyt *b* lineages when they differed by one or more nucleotides. Lineages with no records on public databases were considered novel. All newly-generated sequences were deposited in the GenBank database under accession numbers KX068685–KX068694.

Phylogenetic relationships were inferred using *Plasmodium* lineages found in our study together with lineages deposited in MalAvi that were assigned to morphospecies. A Bayesian phylogenetic tree was constructed using MrBayes 3.2.2 [24] with the GTR + I + G model of nucleotide evolution, following a ModelTest [25] estimation. We ran two Markov chains simultaneously for 5 million generations that were sampled every 1,000 generations. The first 1,250 trees (25%) were discarded as ‘burn-in’ and the remaining trees were used to calculate the posterior probabilities. We did not use an outgroup and the phylogenetic tree was midpoint rooted for presentation as described by Outlaw & Ricklefs [26].

### Blood meal analysis

To identify the blood meal origin of engorged females, a semi-nested PCRs were conducted targeting segments of cyt *b* from both avian and mammalian DNA. Primers and cycling conditions are as described in Sawabe et al. [27]. Primers Avian-3 and Avian-4 were used in the first reaction to detect bird DNA, and the product was used as template in a second reaction containing primers Avian-3 and Avian-8. For the detection of mammalian-derived DNA, the primers Mammalian-1 and Mammalian-7 were used in the first reaction, and the product was used as template in a second reaction with primers Mammalian-7 and Mammalian-2. The PCR mixes had the same reagents and concentrations as described above for the haemosporidian PCRs. All reactions received 1 μl of either genomic DNA or amplicon. DNA extracted from a mosquito containing blood meal from a Humboldt penguin was used as a positive control for the bird detection PCRs, and DNA extracted from a mosquito containing blood meal from a goat as a positive control for the mammalian PCRs. Nuclease-free water was used as a negative control. To test if samples positive for haemosporidian DNA contained traces of host blood, positive abdomen pools were subjected to blood meal PCRs targeting bird and mammalian DNA. PCR products were visualized and sequenced as described above, but here we used only forward primers to identify host species for the sequencing reaction. Sequences were checked for double peaks and the results were compared to sequences in the GenBank database using the basic local alignment search tool (BLAST). The vertebrate species with the highest match to our sequences were considered to be the blood meal source.

### Data analysis

The relative abundance of the 12 most common mosquito species and unidentified *Culex* spp. across successional stages and different seasons were used to construct Bray-Curtis dissimilarity matrices subjected to non-metric multidimensional scaling (NMDS) ordination. Differences between successional stages were tested statistically by a one-way analysis of similarity (ANOSIM). ANOSIM compares average Bray-Curtis dissimilarities within and between groups (e.g. successional stages). It produces an “R” statistics which is positive when average dissimilarities between groups are greater than average dissimilarities within groups. R approaches zero when average dissimilarities between and within groups are similar. R is tested for significance by permuting the grouping variable. These multivariate analyses were conducted with the package *vegan* [28] in R v.3.3.1 [29].

We constructed rank-abundance plots to assess the dominance or evenness of mosquito communities across successional stages separated by season of collection. We used ANCOVA to test for differences in curve slopes between different successional stages. Abundance data were log_10_-transformed for both analyses [30, 31]. These analyses were carried out in the package *BiodiversityR* [32].

## Results

**Fig. 2:**
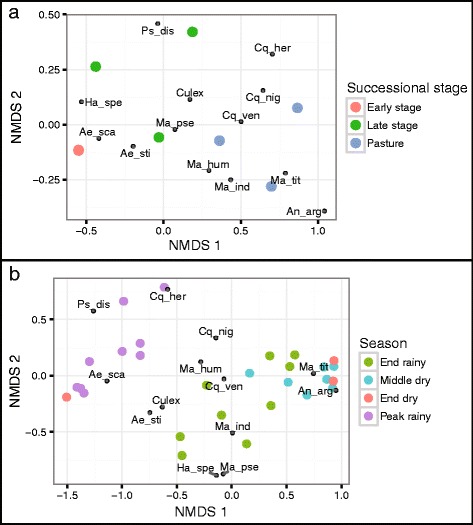
Non-dimensional metric scaling (NMDS) ordination showing mosquito community dissimilarity between successional stages (**a**) and season (**b**). Mosquito communities were significantly dissimilar through all successional stages (ANOSIM: *R* = 0.620, *P* = 0.009) and between the peak of the rainy season and the remaining periods of collection (ANOSIM: *R* = 0.624, *P* = 0.0001). All sampling points from the early stage coalesced in a single point in the graph. *Abbreviations*: Ae_sca, *Ae. scapularis*; Ae_sti, *Ae. stigmaticus*; An_arg, *An. argyritarsis*; Cq_her, *Cq. hermanoi*; Cq_nig, *Cq. nigricans*; Cq_ven, *Cq. venezuelensis*; Culex, *Culex* sp; Ha_spe, *Ha. spegazzinii*; Ma_hum, *Ma. humeralis*; Ma_ind, *Ma. indubitans*; Ma_pse, *Ma. pseudotitillans*; Ma_tit, *Ma. titillans*; Ps_dis, *Ps. discrucians*

We collected 27,110 individual mosquitoes and identified 21,997 to the species level, representing 36 species overall. The distributions of the 12 most common species as well as unidentified *Culex* mosquitoes across successional stages and period of collection are presented in Table 1. Tribe Mansoniini (genera *Mansonia* and *Coquillettidia*) and *Anopheles argyritarsis* were related to pasture areas and accounted for the community dissimilarity between successional stages, as revealed by the NMDS analysis (ANOSIM: *R* = 0.620, *P* = 0.009; Fig. 2a). The exception was that *Mansonia pseudotitillans* was related to both late and early stages of succession. The tribe Aedini (*Aedes*, *Psorophora* and *Haemagogus*) was related to early and late stages and *Culex* spp. were associated with late and pasture stages of succession.

**Table 1:** Abundance of the 12 most common mosquito species and *Culex* sp. sampled in this study

	End rainy season			Middle dry season			End dry season			Peak rainy season			Total per stage			Total
Species	Pasture	Early	Late	Pasture	Early	Late	Pasture	Early	Late	Pasture	Early	Late	Pasture	Early	Late	
*Anopheles argyritarsis*	213	0	2	62	1	1	3	0	0	0	0	0	278	1	3	282
*Aedes scapularis*	264	39	63	8	1	1	0	1	0	2,739	3,507	2,836	3,011	3,548	2,900	9,459
*Aedes stigmaticus*	5	10	1	0	0	0	0	0	0	103	76	95	108	86	96	290
*Haemagogus spegazzinii*	11	17	19	0	0	0	0	0	0	8	33	18	19	50	37	106
*Psorophora discrucians*	6	0	1	0	0	0	0	0	0	609	160	744	615	160	745	1,520
*Coquillettidia hermanoi*	600	6	6	0	0	0	0	0	0	1,674	21	1,258	2,274	27	1,264	3,565
*Cq. nigricans*	42	0	2	1	0	0	0	0	0	51	0	43	94	0	45	139
*Cq. venezuelensis*	43	1	1	0	0	0	0	0	0	21	1	9	64	2	10	76
*Mansonia humeralis*	44	1	0	10	0	0	0	0	0	87	2	170	141	3	170	314
*Ma. indubitans*	56	11	9	0	0	0	0	0	0	0	0	1	56	11	10	77
*Ma. pseudotitillans*	196	35	31	6	0	1	0	0	0	7	3	1	209	38	33	280
*Ma. titillans*	3,375	81	106	345	9	42	49	0	1	635	57	437	4,404	147	586	5,137
*Culex* sp.	0	12	1	0	0	0	0	0	0	148	39	114	148	51	115	314
Total stage/season	4,855	213	242	432	11	45	52	1	1	6,082	3,899	5,726	11,421	4,124	6,014	21,559
Grand total	5,310			488			54			15,707

*Aedes scapularis*, *Coquillettidia hermanoi* and *Psorophora discrucians* accounted for the dissimilarity in mosquito communities in the peak of the rainy season in relation to the other three sampling periods (ANOSIM: *R* = 0.624, *P* = 0.0001; Fig. 2b). On the other hand, *An. argyritarsis* was not detected at the peak of the rainy season.

**Fig. 3:**
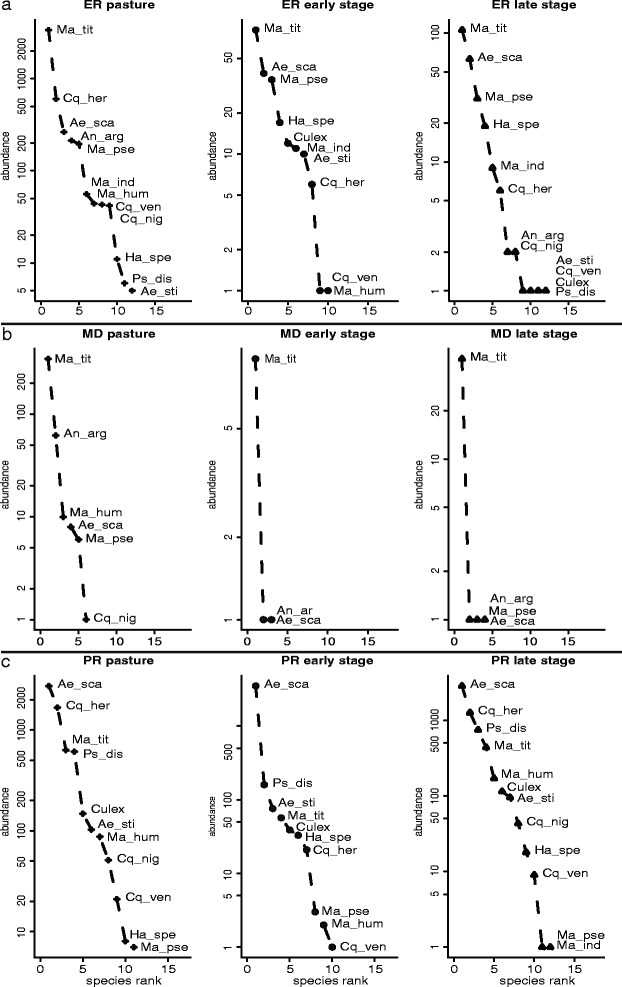
Rank-abundance curves of mosquito species according to successional stages. We did not analyze the end of the dry season due to the small sample sizes. There was no difference in the community evenness between successional stages for all seasons analyzed (ANCOVA: *F*_(1, 74)_ = 0.018, *P* = 0.982). **a** End of the rainy season (*F*_(1,28)_ = 0.615, *P* = 0.548). **b** Middle of the dry season (*F*_(1,7)_ = 0.015, *P* = 0.985). **c** Peak of the rainy season (*F*_(1,28)_ = 1.733, *P* = 0.196). *Abbreviations*: Ae_sca, *Ae. scapularis*; Ae_sti, *Ae. stigmaticus*; An_arg, *An. argyritarsis*; Cq_her, *Cq. hermanoi*; Cq_nig, *Cq. nigricans*; Cq_ven, *Cq. venezuelensis*; Culex, *Culex* sp; Ha_spe, *Ha. spegazzinii*; Ma_hum, *Ma. humeralis*; Ma_ind, *Ma. indubitans*; Ma_pse, *Ma. pseudotitillans*; Ma_tit, *Ma. titillans*; Ps_dis, *Ps. discrucians*

Mosquito abundance was higher in pasture areas, which can be attributed to the distribution of the tribe Mansoniini. *Anopheles argyritarsis* was mainly found in pasture areas (*n* = 278), with only a few specimens in early and late stages of succession (one and three individuals, respectively). We observed a decrease in mosquito abundance in the middle of the dry season, very few mosquitoes captured at the end of the dry season, and an increase of capture rates at the peak of the rainy season. Species dominance shifted between the end and the peak of the rainy season, with *Mansonia titillans* and *Ae. scapularis* representing the most abundant species in each period, respectively. The vast majority of *Ps. discrucians* and *Culex* spp. were captured at the peak of the rainy season, and they were not detected in the dry season. Despite this change in mosquito dominance across successional stages and between the period of sampling, there was no difference in the communities evenness, as indicated by the slopes of our rank-abundance curves (ANCOVA: *F*_(1,74)_ = 0.018, *P* = 0.982; Fig. 3).

We screened 1,913 mosquito pools comprising 17,619 abdomens for the presence of haemosporidians. Two pools of *Ma. titillans* were positive for *Plasmodium* out of 459 tested (total of 4,336 abdomens screened). The lineages retrieved were PAMIT01, detected in two birds in the Mata Seca State Park (Ferreira Jr., unpublished observations) and in a free-living bird in São Paulo Zoo [33], and MaTIT1, a new lineage exhibiting a 97% identity match with the closest related sequences deposited in GenBank. One pool of *Ma. pseudotitillans* (out of 29 samples comprising 224 abdomens) was positive for the *Plasmodium* lineage TUMIG03, which was described in two species of mosquito in the USA [34] and in five species of birds across the American continent, including Brazil [10]. Two pools of *Culex* spp. were positive out of 43 tested (314 abdomens). In one of the *Culex* pools, we detected a single parasite lineage that matched *Plasmodium gallinaceum* (100%). In the other pool of *Culex* spp., we found four different lineages in each of four separate PCRs, with haplotypes differing from 1 to 5 nucleotides when compared to *P. gallinaceum*. In a further sequencing from this sample, we detected six double peaks in the eletrochromatogram, an indication of multiple infections, one of which was a 100% match to *P. gallinaceum* after phasing (i.e. separating out haplotypes from the multiple peaks [35]). A single pool, out of 173 tested pools (1,435 abdomens) of *Ps. discrucians* was found to show a mixed infection of *Haemoproteus* with four double peaks. Phasing the multiple infections enabled us to identify a lineage previously assigned to *Haemoproteus syrnii* found in a screech owl (*Megascops choliba*) from southeastern Brazil [36]. Details regarding detected parasite lineages are shown in Table 2. The remaining mosquito species were negative for haemosporidians; the number of tested pools and the corresponding number of tested abdomens for each species were: *An. argyritarsis* (29; 270); *Ae. scapularis* (673; 6,680); *Ae. stigmaticus* (35; 267); *Hg. spegazzinii* (20; 89); *Cq. hermanoi* (378; 3,440); *Cq. nigricans* (18; 145); *Cq. venezuelensis* (11; 67); *Ma. humeralis* (37; 291); *Ma. indubitans* (7; 61). Overall, four of the positive pools were sampled in pasture areas, while early and late stage areas had one positive sample each.

**Table 2:** Parasite lineages detected in mosquitoes captured at Mata Seca State Park, Brazil

	Successional stage of collection	Season	Lineage name (GenBank acc. No.)	Vertebrate hosts of previous detections (order)	Locations of previous detections (GenBank acc. No.)
*Mansonia titillans*	Late stage	Peak rainy	PAMIT01 (KX068686)	*Leptotila verreauxi* (Columbiformes); *Myiodynastes maculatus* (Passeriformes); *Nycticorax nycticorax* (Pelecaniformes)	MSSP (unpublished observations); São Paulo Zoo (KU057967)
*Mansonia titillans*	Pasture	End rainy	MaTIT01 (KX068685)	New lineage	
*Mansonia pseudotitillans*	Pasture	End rainy	TUMIG03 (KX068687)	*Catharus ustulatus*; *Turdus migratorius*; *Tangara icterocephala*, *Turdus assimilis*; *Turdus amaurochalinus* (Passeriformes)	Alaska, USA (JN792135); Missouri, USA (AF465548); Costa Rica (JN819328); Southeastern Brazil (JX021462)
*Psorophora discrucians*	Early stage	Peak rainy	*Haemoproteus syrnii* PsDIS01 (KX068688)	*Megascops choliba* (Strigiformes)	São Paulo State, Brazil (KJ575554)
*Psorophora discrucians*	Early stage	Peak rainy	PsDIS02 (KX068689)	New lineage	
*Culex* sp.	Pasture	Peak rainy	*P. gallinaceum* (KX068694)	*Gallus gallus domesticus* (Galliformes)	
*Culex* sp.	Pasture	Peak rainy	*P. gallinaceum-*like (KX068690-93)	New lineage	

*Abbreviation*: *MSSP* Mata Seca State Park, unpublished observations

All of the thorax pools corresponding to pools of positive abdomens tested negative for haemosporidians. Furthermore, all positive abdomen pools tested negative for the presence of bird DNA, but we did detect DNA from *Homo sapiens* in two pools (*Ma. titillans* and *Ps. discrucians*). All engorged females were negative for haemosporidians (see tested species below).

**Fig. 4:**
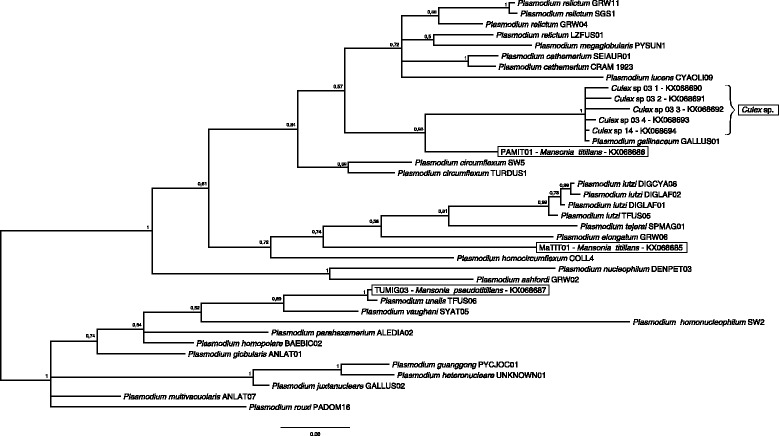
Bayesian phylogenetic tree showing the relationships between haplotypes from this study and parasites with described morphospecies. Lineage names for the morphospecies deposited in MalAvi are provided. Mosquito species together with their associated parasite lineages are shown inside boxes

Phylogenetic analysis revealed that the *Plasmodium* lineages detected in *Mansonia* spp. mosquitoes in the present study are distributed in three different clades (Fig. 4). The genetic distance between the two lineages found in *Ma. titillans* was 2.9% and each sequence differed by 7.5% from the lineage found in *Ma. pseudotitillans.* All *Plasmodium* lineages from *Culex* spp. mosquitoes grouped together with *P. gallinaceum*.

We tested 141 engorged females for the presence of both avian and mammalian DNA: *Ae. scapularis* (*n* = 47); *Hg. spegazzinii* (*n* = 2); *Ps. discrucians* (*n* = 35); *Cq. hermanoi* (*n* = 12); *Cq. nigricans* (*n* = 2); *Cq. venezuelensis* (*n* = 3); *Ma. humeralis* (*n* = 4); *Ma. pseudotitillans* (*n* = 5); and *Ma. titillans* (*n* = 31). All samples showed negative results for avian DNA and 126 samples were positive for the presence of mammalian DNA (89.3%). Two *Ae. scapularis* captured at the peak of the rainy season had blood meal from *Mazama gouazoubira*, a deer species widely distributed in Brazil [37]. One of the engorged individuals was actively collected during the day in the early successional stage, while the other individual was collected using Shannon trap in the pasture area. The remaining samples matched DNA sequences from *Homo sapiens* in mosquitoes captured by both collection methods in all seasons. We did not capture engorged females from *Ae. stigmaticus*, *An. argyritarsis*, *Ma. indubitans* and *Culex* spp., although we can consider these species as attracted to humans.

## Discussion

Our survey revealed *Plasmodium* lineages in the abdomens of the mosquito species *Ma. titillans* and *Ma. pseudotitillans*, suggesting that these species are potential vectors of avian malaria parasites in the SDTF of Brazil. However, detection of *Plasmodium* DNA using molecular methods alone does not confirm transmission; one must also visualize sporozoites in the salivary glands of mosquitoes using microscopy or should conduct experimental infections to confirm true vector identity [38, 39]. Nevertheless, our study represents an important step in identifying vectors of avian malaria parasites in the Neotropics.

The higher abundance of mosquitoes from the tribe Mansoniini in pasture areas may be due to its closer position to the main lake of the Mata Seca State Park. Species from this tribe depend on aquatic macrophytes during their immature life stages to obtain oxygen [18], so this lake probably is the main breeding site for *Mansonia* spp. and *Coquillettidia* spp. The severe dry season in this area drastically reduces breeding sites for species of the tribe Aedini (temporary and small water collections in the soil [18, 40]), but at the peak of the rainy season these species are homogenously distributed across all successional stages. All of the species analyzed in this study utilize the three successional stages to some extent, but several species disappeared during the dry season, showing that seasonality plays a more important role in limiting the dispersion of mosquitoes at MSSP. The putative vectors *Ma. titillans* and *Ma. pseudotitillans* were more abundant in pasture areas, although we understand that other vector species are still do be identified. *Culex* spp. were similarly distributed in the pasture and late successional stages, with lower abundance in the early stages.

The dependency of the tribe Mansoniini on macrophytes suggests that these species may feed upon aquatic birds that gather around those lakes [41]. Chagas et al. [33], working in São Paulo Zoo, detected *Plasmodium* lineage PAMIT01 in a free ranging host *Nycticorax nycticorax*, an aquatic species that inhabits our study area [41]. The same lineage was detected in two bird species sampled at our study area, and one avian host was captured in the same period as the positive mosquitoes we sampled (Ferreira Jr., unpublished observations). We detected the lineage MaTIT01 for the first time. This lineage has a difference of 15 bp in comparison to its closest related *Plasmodium* available on GenBank, but we still do not know which bird species it infects.

The lineage TUMIG03 was detected in passerines from different families across the American continent [42], including southeastern Brazil [10]. Kimura et al. [34] found the same lineage in *Cx. pipiens* and *Cx. restuans* mosquitoes collected in the USA, showing that this lineage may have a wide vector-breadth in parallel with a broad host- and geographical range. The detection of *P. gallinaceum* and closely related lineages in *Culex* spp. is intriguing and should be interpreted with caution. Species of this mosquito genus are the main vectors of avian *Plasmodium* [7] and for this reason *Culex* was included in our analysis despite the lack of identification to the species level. There are no confirmed cases of natural transmission of *Plasmodium gallinaceum* outside of the Asian continent [3], but lineages closely related to this parasite have been detected in wild birds from southeastern Brazil [10] and in captive *Aburria jacutinga*, a bird of the order Galliformes [43]. We mist-netted birds for haemosporidian studies at these same areas and periods, but no *P. gallinaceum*-like parasites were detected (Ferreira Jr., unpublished observations). Future studies in this area should try to access haemosporidians of bird species that are not captured with mist nets, such as Galliformes and aquatic birds, to increase the likelihood of detecting those parasites in their vertebrate hosts.

Culicids have not been confirmed as competent vectors for *Haemoproteus* parasites [44]; however, we did find one abdomen pool of the mosquito *Ps. discrucians* positive for *Haemoproteus*. From this result, we can deduce that this mosquito species uses birds as a blood source, at least occasionally. Experimental work [44, 45] has demonstrated that *Plasmodium* and *Haemoproteus* DNA can be detected in non-vector insects even in the absence of vertebrate DNA, as a result of abortive infections. This scenario explains this finding, and we, therefore, do not include *Psorophora* mosquitoes as putative vectors of *Haemoproteus*.

Sampling methods can influence the community of collected mosquitoes and as a result, the prevalence of haemosporidian parasites [5]. Our trap system relied on LED lamps and human presence as baits, which may have attracted mostly anthropophilic mosquitoes, a result supported by the blood meal analysis of our samples. However, studies testing blood meal source in *Mansonia* spp. and in *Ae. scapularis* in Brazil have shown that these mosquitoes can have ornithophily rates ranging from 20 to 43% [46, 47]. Moreover, Njabo et al. [48] described three species of *Coquillettidia* as putative vectors of avian *Plasmodium* in Cameroon. As species of this genus, together with *Mansonia* spp. and *Ae. scapularis*, constituted 86% of our tested samples, we expected to encounter a higher prevalence of avian haemosporidians. On the other hand, Gager et al. [9] did not detect haemosporidian parasites in 2,760 *Mansonia* and *Coquillettidia* mosquitoes captured in Panama, showing that these genera may not be important vectors of avian haemosporidian parasites in the Neotropics. These contrasting findings show the importance of further studies aiming the identification of avian malaria vectors in South and Central America.

We did not detect avian haemosporidian DNA in thoraces corresponding to positive abdomen pools. Avian malaria vectors can have negative thoraces when corresponding abdomens are positive in experimental and natural conditions [39, 45, 49]. For example, detection of positive thoraces by PCR starts at nine days after experimental infections of competent vectors, with positivity rates varying from 22.2 to 60% afterwards [45]. This suggests that future studies should not disregard the possibility that *Ma. titillans* and *Ma. pseudotitillans* can be vectors of avian haemosporidian parasites just based on our results of negative thorax pools. The same is also true for the potential vectors of *P. gallinaceum* that we identified.

Assessing host-feeding patterns of blood-feeding dipterans can reveal vectors of zoonotic pathogens [50]. Our blood meal analysis revealed that all tested mosquito species feed upon humans, and *Ma. titillans*, *Ma. pseudotitillans*, *Ps. discrucians* and *Culex* spp. mosquitoes also demonstrate ornithophilic behavior. Just recently, antibodies against West Nile virus (WNV) were detected in equines and in chickens in Brazil [51], with the first detection of this virus in humans occurring in 2014 [52]. Other human encephalitis viruses that have birds as reservoirs are transmitted in Brazil, such as the Rocio virus [53], the Mayaro virus [54], the Venezuelan equine encephalitis virus (VEEV) [17] and the Saint Louis encephalitis virus [53]. Moreover, *Ma. titillans* has been detected with WNV in the USA [55] and with VEEV in Mexico [56], addressing to this species a potential role in the transmission of viruses between humans and birds. These results show that potential vectors of important zoonotic diseases use different habitat types in our area of study, and they have access to a high diversity of birds that may act as pathogen reservoirs.

In summary, *Mansonia* mosquitoes can be considered putative vectors of avian *Plasmodium* lineages in in Brazil. We did not detect highly prevalent lineages in our mosquito samples, showing that *M. titillans* and *M. pseudotitillans* are vector candidates of secondary parasite lineages. Indeed, a lineage (MaTIT01) was described here for the first time and the lineage PAMIT01 was detected in two birds out of 63 *Plasmodium* sequences found in captured birds at the same time and place as the mosquitoes (Ferreira Jr., unpublished observations). The lineage TUMIG03 was found in six birds in Brazil [10], but none of those were sampled in Seasonally Dry Tropical Forests. Lacorte et al. [10] detected 33 unique *Plasmodium* lineages in 106 sequences in Seasonally Dry Tropical Forests in Brazil. This highlights the singularity of the haemosporidian community of this ecosystem and reveals the need to identify vectors of the most common *Plasmodium* lineages in Brazil and South America.

## Conclusion

We expanded the list of putative vectors of avian malaria, with the first detections of *Plasmodium* parasites in *Mansonia titillans* and *M. pseudotitillans*. These species are more related to pasture areas, although they were found in all the three different habitats in the area of study. The diversity of avian haemosporidian vectors remain understudied in the Neotropics, and future studies should use different traps to detect new putative vectors of common *Plasmodium* lineages. Furthermore, visual and molecular identification of sporozoites in salivary glands of South American mosquitoes should be attempted in order to elucidate the evolutionary and ecological links between the highly diverse communities of both avian *Plasmodium* spp. and their vectors.

## Abbreviations

SDTF: Seasonally Dry Tropical Forest
MSSP: Mata Seca State Park
cyt *b*: Cytochrome b gene

## References

[1] Knowles SCL, Palinauskas V, Sheldon BC (2010). Chronic malaria infections increase family inequalities and reduce parental fitness: experimental evidence from a wild bird population. J Evol Biol.

[2] Dinhopl N, Nedorost N, Mostegl MM, Weissenbacher-Lang C, Weissenböck H (2015). *In situ* hybridization and sequence analysis reveal an association of *Plasmodium* spp. with mortalities in wild passerine birds in Austria. Parasitol Res.

[3] Valkiūnas G (2005). Avian malaria parasites and other Haemosporidia.

[4] Ricklefs RE (2010). Host-pathogen coevolution, secondary sympatry and species diversification. Philos Trans R Soc B Biol Sci.

[5] Carlson JS, Walther E, TroutFryxell R, Staley S, Tell LA, Sehgal RNM (2015). Identifying avian malaria vectors: sampling methods influence outcomes. Parasit Vectors.

[6] Medeiros MC, Ricklefs RE, Brawn JD, Hamer GL (2015). *Plasmodium* prevalence across avian host species is positively associated with exposure to mosquito vectors. Parasitology.

[7] Santiago-Alarcon D, Palinauskas V, Schaefer HM (2012). Diptera vectors of avian haemosporidian parasites: untangling parasite life cycles and their taxonomy. Biol Rev.

[8] Rueda LM (2008). Global diversity of mosquitoes (Insecta: Diptera: Culicidae) in freshwater. Hydrobiologia.

[9] Gager AB, Del Rosario LJ, Dearborn DC, Bermingham E (2008). Do mosquitoes filter the access of *Plasmodium* cytochrome *b* lineages to an avian host?. Mol Ecol.

[10] Lacorte GA, Félix GMF, Pinheiro RRB, Chaves AV, Almeida-Neto G, Neves FS (2013). Exploring the diversity and distribution of neotropical avian malaria parasites - a molecular survey from southeast Brazil. PLoS One.

[11] Villar CM, Bryan AL, Lance SL, Braga EM, Congrains C, Del Lama SN (2013). Blood parasites in nestlings of wood stork populations from three regions of the American continent. J Parasitol.

[12] Roos FL, Belo NO, Silveira P, Braga EM (2015). Prevalence and diversity of avian malaria parasites in migratory Black Skimmers (*Rynchops niger*, Laridae, Charadriiformes) from the Brazilian Amazon Basin. Parasitol Res.

[13] Belo NO, Pinheiro RT, Reis ES, Ricklefs RE, Braga ÉM (2011). Prevalence and lineage diversity of avian haemosporidians from three distinct cerrado habitats in Brazil. PLoS One.

[14] Fecchio A, Lima MR, Svensson-Coelho M, Marini MÂ, Ricklefs RE (2013). Structure and organization of an avian haemosporidian assemblage in a Neotropical savanna in Brazil. Parasitology.

[15] Alvares CA, Stape JL, Sentelhas PC, de Moraes G, Leonardo J, Sparovek G (2013). Köppen’s climate classification map for Brazil. Meteorol Z.

[16] Madeira BG, Espírito-Santo MM, Neto SD, Nunes YRF (2009). Arturo Sánchez Azofeifa G, Fernandes GW, et al. Changes in tree and liana communities along a successional gradient in a tropical dry forest in south-eastern Brazil. Plant Ecol.

[17] Consoli RAGB, de Oliveira RL (1994). Principais mosquitos de importância sanitária no Brasil.

[18] Forratini OP (2002). Culicidologia Médica: Identificação, Biologia, Epidemiologia Vol. 2.

[19] Reinert JF (1975). Mosquito generic and subgeneric abbreviations (Diptera: Culicidae). Mosq Syst.

[20] Hellgren O, Waldenström J, Bensch S (2004). A new PCR assay for simultaneous studies of *Leucocytozoon*, *Plasmodium*, and *Haemoproteus* from avian blood. J Parasitol.

[21] Bensch S, Stjernman M, Hasselquist D, Ostman O, Hansson B, Westerdahl H (2000). Host specificity in avian blood parasites: a study of *Plasmodium* and *Haemoproteus* mitochondrial DNA amplified from birds. Proc R Soc B Biol Sci.

[22] Tamura K, Stecher G, Peterson D, Filipski A, Kumar S (2013). MEGA6: molecular evolutionary genetics analysis version 6.0. Mol Biol Evol.

[23] Bensch S, Hellgren O, Pérez-Tris J (2009). MalAvi: a public database of malaria parasites and related haemosporidians in avian hosts based on mitochondrial cytochrome *b* lineages. Mol Ecol Resour.

[24] Ronquist F, Huelsenbeck JP (2003). MrBayes 3: Bayesian phylogenetic inference under mixed models. Bioinformatics.

[25] Posada D, Crandall KA (1998). MODELTEST: testing the model of DNA substitution. Bioinformatics.

[26] Outlaw DC, Ricklefs RE (2011). Rerooting the evolutionary tree of malaria parasites. Proc Natl Acad Sci USA.

[27] Sawabe K, Isawa H, Hoshino K, Sasaki T, Roychoudhury S, Higa Y (2010). Host-feeding habits of *Culex pipiens* and *Aedes albopictus* (Diptera: Culicidae) collected at the urban and suburban residential areas of Japan. J Med Entomol.

[28] Oksanen J, Blanchet FG, Friendly M, Kindt R, Legendre P, McGlinn D, et al. vegan: Community ecology package [Internet]. 2016. version no. 2.4-1, 291 pages. Available from: https://cran.r-project.org/web/packages/vegan/index.html.

[29] R Development Core Team (2016). R: A Language and Environment for Statistical Computing [Internet].

[30] Magurran AE (2003). Measuring biological diversity.

[31] Abella-Medrano CA, Ibáñez-Bernal S, MacGregor-Fors I, Santiago-Alarcon D (2015). Spatiotemporal variation of mosquito diversity (Diptera: Culicidae) at places with different land-use types within a neotropical montane cloud forest matrix. Parasit Vectors.

[32] Kindt R (2016). BiodiversityR: package for community ecology and suitability analysis [internet].

[33] Chagas CRF, Guimarães Lde O, Monteiro EF, Valkiūnas G, Katayama MV, Santos SV (2015). Hemosporidian parasites of free-living birds in the São Paulo Zoo, Brazil. Parasitol Res.

[34] Kimura M, Darbro JM, Harrington LC (2010). Avian malaria parasites share congeneric mosquito vectors. J Parasitol.

[35] Matthews AE, Ellis VA, Hanson AA, Roberts JR, Ricklefs RE, Collins MD (2016). Avian haemosporidian prevalence and its relationship to host life histories in eastern Tennessee. J Ornithol.

[36] Vanstreels RET, Kolesnikovas CKM, Sandri S, Silveira P, Belo NO, Ferreira Junior FC (2014). Outbreak of avian malaria associated to multiple species of *Plasmodium* in Magellanic penguins undergoing rehabilitation in southern Brazil. PLoS One.

[37] Duarte JMB, Vogliotti A, dos Santos Zanetti E, de Oliveira ML, Tiepolo LM, Rodrigues LF, et al. Avaliação do risco de extinção do veado-catingueiro *Mazama gouazoubira* G. Fischer [von Waldhein], 1814, no Brasil. Biodiversidade Bras. 2012;3:50–58.

[38] Valkiūnas G (2011). Haemosporidian vector research: marriage of molecular and microscopical approaches is essential. Mol Ecol.

[39] Kim K, Tsuda Y (2015). Sporogony and sporozoite rates of avian malaria parasites in wild *Culex pipiens* pallens and *C inatomii* in Japan. Parasit Vectors.

[40] Lourenço-de-Oliveira R, Heyden R, da Silva TF (1986). Some aspects of the ecology of mosquitoes (Diptera, Culicidae) of an area of plains (granjas Calábria), in Jacarepaguá, Rio de Janeiro: V. Breeding places. Mem Inst Oswaldo Cruz.

[41] Dornelas AAF, de Paula DC, Santo MME, Azofeifa GS, Leite LO (2012). Avifauna of the Mata Seca State Park, north of Minas Gerais. Rev Bras Ornitol-Braz J Ornithol.

[42] Dodge M, Guers SL, Sekercioğlu ÇH, Sehgal RNM (2013). North American transmission of hemosporidian parasites in the Swainson’s thrush (*Catharus ustulatus*), a migratory songbird. J Parasitol.

[43] Motta ROC, Romero Marques MV, Ferreira Junior FC, Andery Dde A, Horta RS, Peixoto RB (2013). Does haemosporidian infection affect hematological and biochemical profiles of the endangered Black-fronted piping-guan (*Aburria jacutinga*)?. Peer J.

[44] Valkiūnas G, Kazlauskienė R, Bernotienė R, Palinauskas V, Iezhova TA (2013). Abortive long-lasting sporogony of two *Haemoproteus* species (Haemosporida, Haemoproteidae) in the mosquito *Ochlerotatus cantans*, with perspectives on haemosporidian vector research. Parasitol Res.

[45] Kim KS, Tsuda Y, Sasaki T, Kobayashi M, Hirota Y (2009). Mosquito blood-meal analysis for avian malaria study in wild bird communities: laboratory verification and application to *Culex sasai* (Diptera: Culicidae) collected in Tokyo, Japan. Parasitol Res.

[46] Lorosa ES, Faria MS, De Oliveira LCM, Alencar J, Marcondes CB (2010). Blood meal identification of selected mosquitoes in Rio De Janeiro, Brazil. J Am Mosq Control Assoc.

[47] dos Santos SJ, Alencar J, Costa JM, Seixas-Lorosa E, Guimarães AÉ (2012). Feeding patterns of mosquitoes (Diptera: Culicidae) in six Brazilian environmental preservation areas. J Vector Ecol.

[48] Njabo KY, Cornel AJ, Bonneaud C, Toffelmier E, Sehgal RNM, Valkiūnas G (2011). Nonspecific patterns of vector, host and avian malaria parasite associations in a central African rainforest. Mol Ecol.

[49] Kazlauskienė R, Bernotienė R, Palinauskas V, Iezhova TA, Valkiūnas G (2013). *Plasmodium relictum* (lineages pSGS1 and pGRW11): complete synchronous sporogony in mosquitoes *Culex pipiens pipiens*. Exp Parasitol.

[50] Santiago-Alarcon D, Havelka P, Pineda E, Segelbacher G, Schaefer HM (2013). Urban forests as hubs for novel zoonosis: blood meal analysis, seasonal variation in *Culicoides* (Diptera: Ceratopogonidae) vectors, and avian haemosporidians. Parasitology.

[51] Melandri V, Guimarães AÉ, Komar N, Nogueira ML, Mondini A, Fernandez-Sesma A (2012). Serological detection of West Nile virus in horses and chicken from Pantanal, Brazil. Mem Inst Oswaldo Cruz.

[52] Vieira MACS, Romano APM, Borba AS, Silva EVP, Chiang JO, Eulálio KD (2015). West Nile virus encephalitis: the first human case recorded in Brazil. Am J Trop Med Hyg.

[53] Ferreira IB, Pereira LE, Rocco IM, Marti AT, de Souza LT, Iversson LB (1994). Surveillance of arbovirus infections in the atlantic forest region, State of São Paulo, Brazil. I. Detection of hemagglutination-inhibiting antibodies in wild birds between 1978 and 1990. Rev Inst Med Trop Sao Paulo.

[54] Figueiredo LTM (2007). Emergent arboviruses in Brazil. Rev Soc Bras Med Trop.

[55] Unlu I, Kramer WL, Roy AF, Foil LD (2010). Detection of West Nile virus RNA in mosquitoes and identification of mosquito blood meals collected at alligator farms in Louisiana. J Med Entomol.

[56] Adams AP, Navarro-Lopez R, Ramirez-Aguilar FJ, Lopez-Gonzalez I, Leal G, Flores-Mayorga JM (2012). Venezuelan equine encephalitis virus activity in the gulf coast region of Mexico, 2003–2010. PLoS Negl Trop Dis.

